# Effects of *Corcyra cephalonica* Egg Consumption on Population Fitness and Reproduction of the Whitefly Predator *Serangium japonicum* (Coleoptera: Coccinellidae)

**DOI:** 10.3390/insects17010062

**Published:** 2026-01-03

**Authors:** Jianfeng Liang, Jing Peng, Huiyi Cao, Yuxia Hu, Muhammad Irfan Ullah, Shaukat Ali, Xingmin Wang

**Affiliations:** 1 College of Plant Protection, South China Agricultural University, Guangzhou 510642, China; liangjf628@163.com (J.L.); pengjing17@scau.edu.cn (J.P.); 15976346478@163.com (H.C.); hyx422@stu.scau.edu.cn (Y.H.); aliscau@scau.edu.cn (S.A.); 2 Engineering Research Center of Biological Control, Ministry of Education and Guangdong Province, South China Agricultural University, Guangzhou 510642, China; 3 Department of Entomology, University of Sargodha, Sargodha 40100, Pakistan; muhammad.irfanullah@uos.edu.pk

**Keywords:** predatory natural enemies, large-scale production, *Bemisia tabaci*, alternative prey, ovary development

## Abstract

Ladybird beetle, *Serangium japonicum* (Coleoptera: Coccinellidae), is an important predatory natural enemy of whiteflies, and its mass rearing is crucial for biological control. This study evaluated the suitability of *Corcyra cephalonica* (Lepidoptera: Pyralidae) eggs as an alternative diet for adult *S. japonicum* by directly comparing it to the natural prey, *Bemisia tabaci* (Hemiptera: Aleyrodidae) nymphs. Results showed that, compared to a *B. tabaci* diet, feeding on *C. cephalonica* eggs supported normal development and significantly extended the lifespan of adult *S. japonicum*, without compromising offspring quality (hatching rate, development, survival, or predatory capacity). However, the moth egg diet significantly impaired reproduction, causing delayed ovarian development, reduced vitellogenesis, and altered gene expression: downregulation of methoprene-tolerant, Juvenile hormone acid O-methyltransferase, Vitellogenin, and Vitellogenin receptor, and upregulation of Juvenile hormone esterase and Copper/zinc superoxide dismutase. Practically, this work defines *C. cephalonica* eggs as a suboptimal but viable supplementary diet for colony maintenance, but unsuitable as a sole diet for mass-rearing reproductively robust populations. Our findings explain the physiological and molecular mechanisms underlying the “reproduction–lifespan trade-off” in *S. japonicum* induced by feeding on *C. cephalonica* eggs, providing a mechanistic basis for its rational application in the mass production of natural enemies.

## Simple Summary

Ladybird beetle, *Serangium japonicum,* is an effective natural enemy of whiteflies in China. *Serangium japonicum* has shown promising results in field, but its mass-production needs a suitable and affordable artificial diet. This study investigates the feasibility of using rice moth (*Corcyra cephalonica*) as an alternative food source for *S. japonicum* adults. We found that while this diet allowed the beetles to develop normally with longer life periods, it significantly reduced their ability to reproduce by delaying ovary development as well as affecting the activity of key reproduction related genes. Our findings explain the trade-off between a longer life period and lower reproduction when feeding on this alternative diet. These findings provide crucial information for optimizing the mass production of this natural enemy for effective and sustainable pest control.

## 1. Introduction

As crucial natural regulatory agents in ecosystems, insect natural enemies play a significant role in the biological control of agricultural pests. With the advancement of green agriculture, the industrialization of insect natural enemies has encountered important opportunities [1]. In Europe as well as the United States, several insect natural enemies (such as *Delphastus catalinae* (Coleoptera: Coccinellidae), *Chrysoperla rufilabris* (Neuroptera: Chrysopidae), and *Macrolophus caliginosus* (Hemiptera: Miridae)) have been produced commercially. Artificial media can effectively replace insect eggs for the large-scale production of parasitic natural enemies [2,3]. However, the commercial mass production of predatory natural enemies still faces numerous challenges, with mass rearing and long-term storage remaining bottleneck issues restricting their application [4,5].

The commercialization of insect natural enemies encompasses four core steps including mass production, storage, transportation, and release. Due to complex tri-trophic interactions, this supply chain is often time-consuming and labor-intensive, making large-scale production difficult. Furthermore, low shelf life of natural enemies is a major obstacle during storage and transportation. The immature stages of parasitic natural enemies typically develop within their hosts, resulting in lower environmental requirements and better storage tolerance. In contrast, the eggs of predatory natural enemies are prone to hatching during transport and active larvae require continuous food and space which can significantly increase their management complexity [6]. Techniques like low-temperature storage can extend shelf life by reducing metabolism [7,8], but prolonged refrigeration often leads to significant declines in survival, fecundity, and predation capacity [9,10]. Most insect natural enemies are still reared using the traditional “host plant-prey-natural enemy” model, which is associated with high costs, long cycles, and difficult-to-control instability during rearing [11]. Using artificial diets or alternative prey to fulfill the nutritional requirements of natural enemies can simplify the rearing process [12,13,14]. However, artificial diets often fail to fully replicate the physical state and nutritional composition of natural prey, leading to different issues (reduced eclosion rates, survival, and reproductive capacity in natural enemies) [15]. In comparison, alternative diets (*Corcyra cephalonica* eggs, *Ephestia kuehniella* (Lepidoptera: Pyralidae) eggs, *Trichogramma* pupae, aphids, and eggs or larvae of other lepidopteran pests) offer more comprehensive nutrition and better align with the natural dietary preferences of predatory ladybirds, often yielding better results than semi-artificial diets [16]. However, the adaptability to alternative prey varies significantly among different ladybird species. For example, while *Coccinella septempunctata* (Coleoptera: Coccinellidae) can complete larval development on psyllid prey, it fails to oviposit when fed such prey [16]. *Harmonia axyridis* (Coleoptera: Coccinellidae) can survive on *Tuberolachnus salignus* (Hemiptera: Aphididae) but exhibits prolonged development, reduced body weight, and increased mortality in immature stages [17]. Similarly, *C. cephalonica* eggs have been successfully used as alternative prey for rearing *Xylocoris flavipes* (Hemiptera: Anthocoridae) [18], but when fed to larvae of *H. axyridis* and *Propylea japonica*, all early-instar larvae died [19]. Notably, *Delphastus catalinae* (Coleoptera: Coccinellidae) resumed oviposition within four days after being switched from a 35-day diet of *C. cephalonica* eggs to natural prey, with no significant difference in total egg production [20], indicating the potential application of alternative prey under specific conditions.

*Serangium japonicum* (Coleoptera: Coccinellidae), widely distributed across China, is a dominant predator of whitefly pests. Both its larvae and adults exhibit strong predatory capacity by effectively suppressing pest populations [11]. *S. japonicum* possesses characteristics such as high predation rate, fecundity, short reproductive cycle, and long lifespan [21], indicating promising potential for industrial application. The mass production of *S. japonicum*, essential for field releases is constrained by its dependency on a continuous supply of *B. tabaci*. To overcome this limitation, exploring nutritionally adequate and logistically feasible alternative foods is of high priority. Notably, eggs of the rice moth (*Corcyra cephalonica*), a perennial pest of stored grains like rice and bran, represent a widely adopted and accessible factitious food source in the commercial production of many beneficial arthropods [22]. Preliminary studies have shown that feeding *S. japonicum* larvae on *C. cephalonica* eggs supports normal development and survival [23]. Given that the suitability of prey may vary across different life stages of predatory ladybirds, it is necessary to separately evaluate the effects of alternative prey on different life history parameters of larval as well as adult stages [23,24]. Therefore, this study aims at investigating the effects of feeding *S. japonicum* on *C. cephalonica* eggs on population fitness, ovarian development, and the expression of reproductive-related genes. The main objective of this study is to elucidate the potential impacts of *C. cephalonica* eggs as an alternative diet on female reproduction in *S. japonicum* from both physiological and molecular perspectives, thereby providing a theoretical basis for selecting alternative prey for natural enemy ladybirds and offering new insights for achieving mass rearing of *S. japonicum*.

## 2. Materials and Methods

### 2.1. Insect Rearing

*Bemisia tabaci* MEAM1 (Hemiptera: Aleyrodidae) was reared on healthy, clean cotton seedlings. Stock colonies of *S. japonicum* were fed on cotton seedlings infested with *B. tabaci* nymphs. All insects were reared in an insect rearing room under controlled conditions: temperature 26 ± 2 °C, relative humidity 70 ± 5%, and a photoperiod of 14 L:10 D. *S. japonicum* pupae were collected daily from the stock colony and transferred into petri dishes (6 cm diameter), which were then placed in an RXZ-436E-LED intelligent artificial climate chamber (Ningbo Jiangnan Instrument Factory, Ningbo, China). The environmental parameters within the climate chamber were consistent with those of the rearing room. Adult emergence was monitored daily, and newly emerged adults from the same day were collected into new Petri dishes for subsequent experiments. *C. cephalonica* eggs used in the experiments were purchased from Guangzhou Yuefeng Biological Control Technology Co., Ltd. Fresh eggs were inactivated prior to use by exposure to germicidal ultraviolet (UV) irradiation (wavelength: 253.7 nm, UV-C spectrum) for 30 min using a standard laboratory UV lamp. Following UV treatment, eggs were immediately used for feeding.

### 2.2. Assessment of the Effects of Different Diets on the Population Fitness of S. japonicum

Newly emerged *S. japonicum* adults were sexed and paired. Each pair was housed in a petri dish. Two dietary treatments were established: (1) *B. tabaci* nymphs, and (2) *C. cephalonica* eggs. To standardize the feeding substrate, both prey types were inoculated to cotton leaves. Each treatment consisted of 10 replicates. Diets were replaced daily. Adult survival and oviposition were recorded.

Eggs laid by females from both treatment groups (designated as the F_1_ generation) were collected. To ensure developmental synchrony, fresh eggs laid during the peak oviposition period (within a 24-h interval) were collected and used for further experiments. Egg hatching was observed and recorded every 12 h. Newly hatched larvae were individually transferred to new petri dishes using a fine camel hair brush (No. 001) and were fed on *B. tabaci* nymphs daily until pupation. The egg hatch rate, generation developmental duration, and generation survival rate of the F_1_ generation from both groups were recorded and compared. For each treatment, 30 eggs were observed, with 3 replicates. To evaluate the predatory capacity of the F_1_ larvae, when larvae reached the 4th instar, they were individually placed in Petri dishes and starved for 12 h. Subsequently, a cotton leaf bearing 100 fourth-instar *B. tabaci* nymphs was introduced into each dish. The number of remaining whitefly nymphs in each dish was recorded after 24 h. This assay included 10 replicates per treatment.

### 2.3. Female Ovary Dissection and Body Weight Measurement

Newly emerged *S. japonicum* adults were sexed and paired, then divided into two groups, either *B. tabaci* nymphs or *C. cephalonica* eggs for periods ranging from 1 to 20 days. At different ages post-emergence, females were distinguished under a Stemi305 stereomicroscope (Carl Zeiss AG, Oberkochen, Germany). Female beetles (*n* = 10 per time point) were randomly selected, and their body weight was measured using a precision balance (accuracy 0.0001 g). This process was performed three times. Subsequently, dissection was performed. The elytra and hindwings of the female beetles were removed, and the insects were placed in a dissection dish containing 0.9% NaCl solution. Using an insect pin (0.38 mm), an incision was made along the dorsal side of the abdomen. The peritoneal membrane was removed, and fat bodies and other tissues were carefully cleared to expose and completely extract the ovaries. The extracted ovaries were placed on a glass slide with a drop of saline. The overall morphology of the ovaries was photographed using a Stemi 508 microscope camera (Carl Zeiss AG, Oberkochen, Germany). The ovarioles were then carefully arranged in their natural extended state, covered with a coverslip applying gentle pressure, and their lengths were measured using AxioVision Rel. 4.8 software. Three females were dissected for each age group.

### 2.4. Measurement of Female Reproduction-Related Gene Expression

Newly emerged *S. japonicum* females that had been fed either *C. cephalonica* eggs or *B. tabaci* nymphs for 20 days were collected. Three biological replicates were prepared per diet treatment, with each replicate consisting of a pooled sample of 10 individuals. The samples were immediately frozen in liquid nitrogen for 15 s, and then transferred to a −80 °C ultra-low temperature freezer for subsequent RNA extraction. Total RNA was extracted from the samples using Trizol reagent (Accurate Biology, Changsha, Hunan, China). RNA concentration and the A260/A280 ratio were determined using a NanoDrop 2000 spectrophotometer (Thermo Fisher Scientific, Bremen, Germany). The ratios for all samples fell within the range of 1.8–2.0, indicating acceptable RNA purity. RNA was reverse transcribed into cDNA using the PrimeScript™ RT Reagent Kit (RR047A, Takara Bio Inc., Kusatsu, Shiga). The expression levels of the following key reproduction-related genes were detected using real-time quantitative PCR: methoprene-tolerant (*Met*, Juvenile hormone receptor), Juvenile hormone acid O-methyltransferase (*JHAMT*), Juvenile hormone esterase (*JHE*), Vitellogenin (*Vg*), Vitellogenin receptor (*VgR*), and Copper/zinc superoxide dismutase (*Cu/Zn SOD*). The *β*-actin gene was used as the internal reference gene. Primers were designed using Primer Premier 6 (PremierBiosoft, Palo Alto, CA, USA); primer names and sequences are listed in Table 1. The qPCR reaction mixture (10 µL total volume) consisted of: 5 µL SYBR Green PCR Master Mix (Accurate Biology, Changsha, Hunan, China), 2 µL cDNA, 0.5 µL each of forward and reverse primers, and 2 µL ddH_2_O. Amplification was performed using a CFX Connect™ Real-Time System (Bio-Rad, Hercules, CA, USA) with the following program: initial denaturation at 95 °C for 3 min, followed by 39 cycles of denaturation at 95 °C for 10 s, annealing at 60 °C for 20 s, and extension at 72 °C for 30 s. Relative expression levels were calculated using the 2^−ΔΔCt^ method [25].

### 2.5. Statistical Analyses

Data were analyzed using SPSS 23.0 software (IBM, Armonk, NY, USA). Statistical comparisons between the two groups for each measured parameter were performed independently. The choice of test was guided by diagnostic checks: data were first assessed for normality (Shapiro-Wilk test). Non-normal data were analyzed using the Mann-Whitney U test. For normal data, variance homogeneity (Levene’s test) was then evaluated; data with equal variances were compared using the standard independent samples *t*-test, while those with unequal variances were analyzed with Welch’s *t*-test. All data are presented as mean ± standard error (SE). Figures were generated using GraphPad Prism 8.0 (GraphPad, San Diego, CA, USA).

## 3. Results

### 3.1. Effects of Feeding on C. cephalonica Eggs on the Population Fitness of S. japonicum

**Figure 1:**
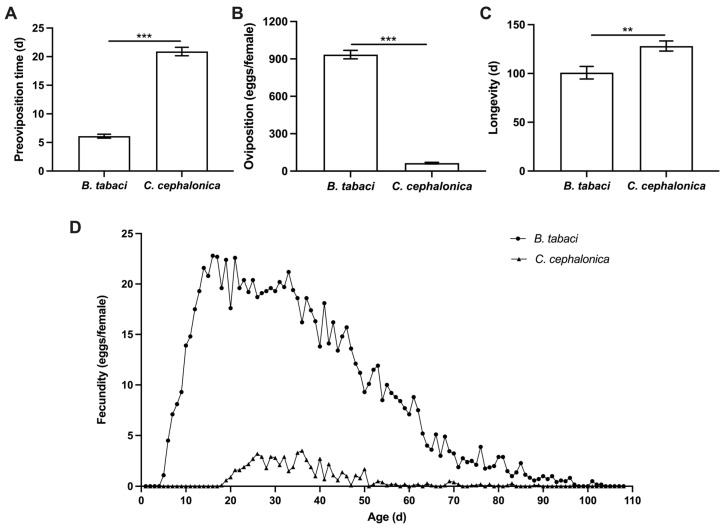
Effects of feeding on *C. cephalonica* eggs on the population fitness of *S. japonicum* adults. (**A**): Preoviposition time. (**B**): Oviposition. (**C**): Longevity. (**D**): Average daily oviposition. All data are presented as mean ± standard error (SE) (*n* = 10). Asterisks indicate statistically significant differences: ** *p* < 0.01, *** *p* < 0.001.

Compared to the group fed *B. tabaci* nymphs, female *S. japonicum* fed *C. cephalonica* eggs exhibited a significantly longer pre-oviposition period, which increased from 6.10 ± 0.35 days to 20.90 ± 0.74 days (*t* = 18.157, d.f. = 12.822, *p* < 0.001). Total fecundity of *S. japonicum* adults feeding *C. cephalonica* eggs was significantly lower (64.30 ± 6.48 eggs/female) than those feeding on *B. tabaci* nymphs (934.70 ± 34.20 eggs/female) (*t* = 25.004, d.f. = 9.645, *p* < 0.001). In contrast, their adult longevity was significantly extended (128.10 ± 5.18 vs. 100.80 ± 6.49 days; *t* = 3.289, df = 18, *p* = 0.004). Analysis of daily mean oviposition dynamics (Figure 1) revealed that *S. japonicum* fed *B. tabaci* nymphs rapidly reached peak daily egg production after the pre-oviposition period, with the peak oviposition period concentrated between 20 and 35 days of age, after which daily egg production gradually declined until death. In contrast, females fed *C. cephalonica* eggs maintained a consistently low level of daily egg production. Their peak oviposition occurred later (30–40 days of age), with a maximum daily output not exceeding 5 eggs, followed by only sporadic oviposition until death.

**Figure 2:**
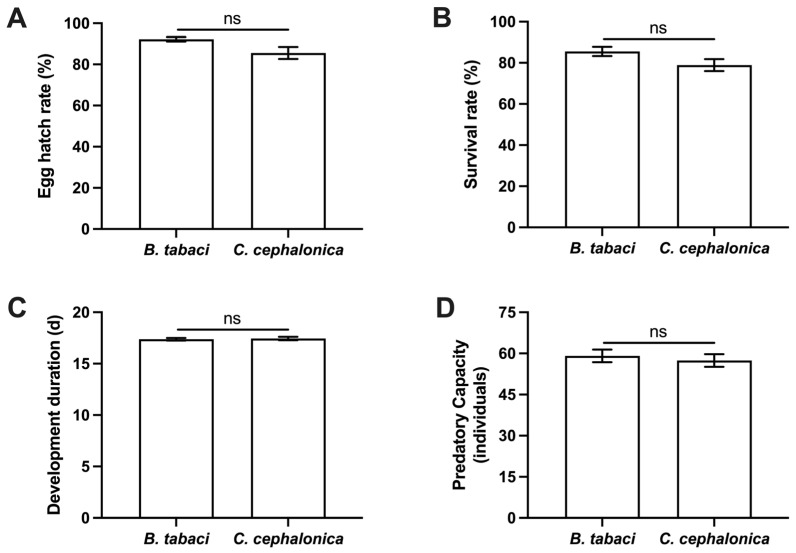
Effects of feeding on *C. cephalonica* eggs on the growth and predation of the first generation of *S. japanicum.* (**A**): Egg hatch rate. (**B**): Survival rate. (**C**): Developmental duration. (**D**): Predatory capacity of fourth instar larvae against fourth-instar nymphs of *B. tabaci*. All data are presented as mean ± standard error (SE). For panels (**A**–**C**), data are derived from three biological replicates, each containing 30 individuals. For panel (**D**), *n* = 10. The abbreviation “ns” denotes no significant difference between groups.

Feeding on *C. cephalonica* eggs had no significant effects on the egg hatch rate, generation survival rate, generation developmental duration, or the predation capacity of F1 fourth-instar larvae on *B. tabaci* nymphs (Figure 2). Specifically, the egg hatching rate of the F1 generation from the *C. cephalonica* egg group (92.22 ± 1.11%) was not significantly different from those feeding on *B. tabaci* (85.56 ± 2.94%) (Mann-Whitney U test: U = 0.500, Z = −1.798, *p* = 0.100). Similarly, the F1 generation survival rate was also comparable between the two groups (85.55 ± 2.22% vs. 78.89 ± 2.94%, U = 1.000, Z = −1.623, *p* = 0.200). Regarding development, the generation duration of the F_1_ generation from the *C. cephalonica* egg group was 17.37 ± 0.12 days, essentially identical to those feeding on *B. tabaci* (17.44 ± 0.16 days) (*t* = 0.354, d.f. = 4, *p* = 0.741). Furthermore, the number of fourth-instar *B. tabaci* nymphs consumed within 24 h by F_1_ fourth-instar larvae from both groups was 59.1 ± 2.29 and 57.4 ± 2.30, respectively, again showing no significant difference (*t* = 0.524, d.f. = 18, *p* = 0.607).

### 3.2. Effects of Feeding on C. cephalonica Eggs on Female Ovary Development and Body Weight of S. japonicum

The ovaries of *S. japonicum* are tree-like, with each ovary consisting of four ovarioles connected by terminal filaments. Their number is constant, but development is not entirely synchronous. Based on ovariole morphology and length, vitellogenesis in the ovarioles and the occurrence of mature oocytes, the ovarian development of *S. japonicum* was classified to 6 stages [26]. Stage 0: Ovariole length 0.26–0.41 mm. Characteristic of newly emerged, unfed adults. Ovaries undeveloped; ovarioles slender and straight, without follicle formation. Stage 1: Ovariole length 0.34–0.54 mm. Ovarioles show indentations, appearing gourd-shaped and enveloped by tracheoles, but not differentiated into distinct follicles. Stage 2: Ovariole length 0.49–0.67 mm. Ovarioles elongate, the basic ovarian framework is formed, but no vitellin deposition is observed. Stage 3: Ovariole length 0.46–0.65 mm. Vitellogenesis begins in the first follicle. Stage 4: Ovariole length 0.53–1.08 mm. Subsequent follicles enlarge progressively or show vitellin deposition, but no mature oocytes are present. Stage 5: Ovariole length 0.51–1.51 mm. The oocyte in the first follicle is mature, and the chorion is formed. Stage 6: Ovariole length 0.62–1.54 mm. Ovaries fully developed; mature eggs visible in the lateral or common oviduct.

**Figure 3:**
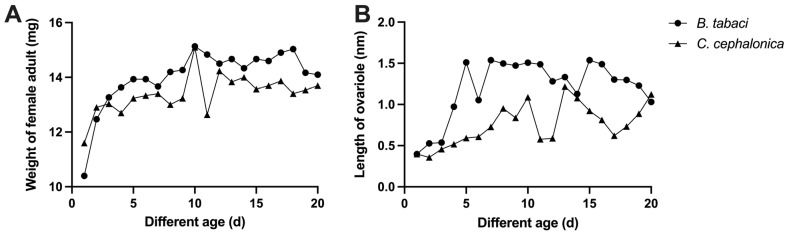
Changes in the length of ovariole and weight of *S. japonicum* females feeding on different prey. (**A**) Changes in weight from 1 to 20 days of age. (**B**) Changes in ovariole length from 1 to 20 days of age.

**Figure 4:**
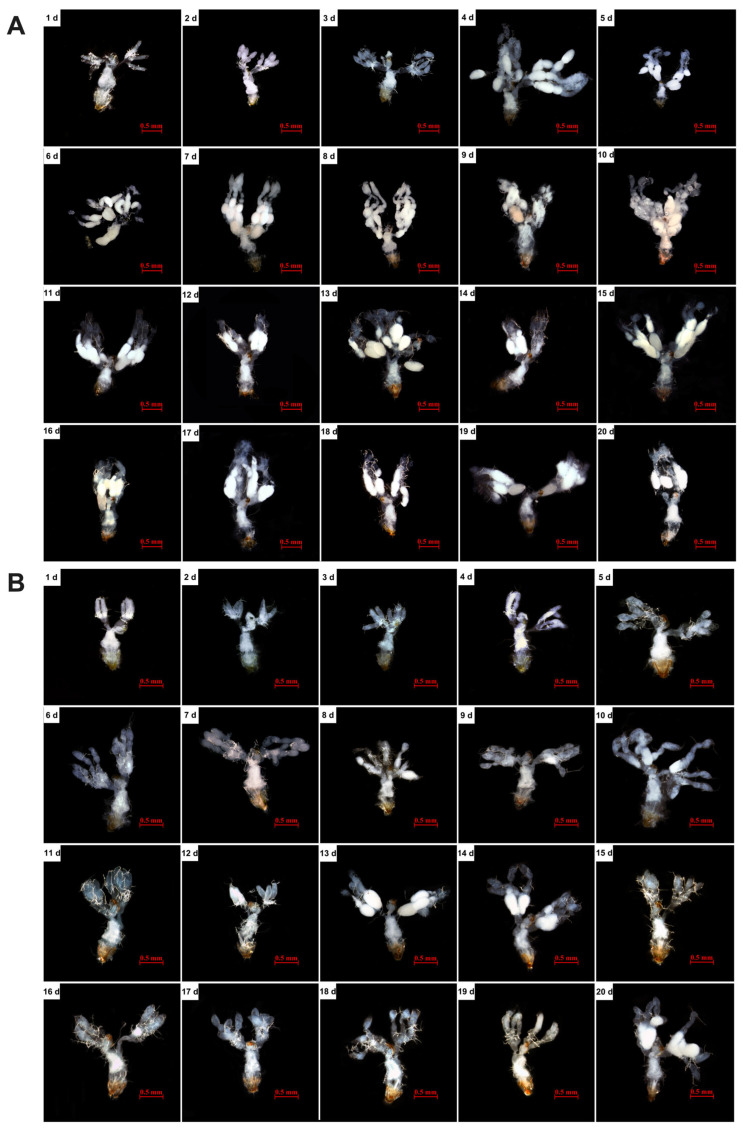
Effects of feeding on *C. cephalonica* eggs on ovarian development of females of *S. japonicum*. (**A**) Ovarian development of females feeding on *B. tabaci* nymphs from 1 to 20 days of age. (**B**) Ovarian development of females feeding on *C. cephalonica* eggs from 1 to 20 days of age.

As shown in Figure 3 and Table 2, feeding on *C. cephalonica* eggs did not significantly effect the development of female ovarioles (*t* = 2.584, df = 4, *p* = 0.061) but impaired the processes of vitellogenesis and oocyte maturation, thereby delaying overall ovarian development and maturation (Figure 4). Females fed on *B. tabaci* nymphs exhibited rapid ovarian development, with mature oocytes (reaching Stage 5) appearing as early as day 5 post-emergence, and most individuals achieved fully developed ovaries (Stage 6) by day 6. In contrast, ovarian development was severely delayed in females fed *C. cephalonica* eggs. Their ovarioles had only completed basic structural development (stagnating at Stage 2) by day 6, with no vitellin accumulation internally. Vitellogenesis began slowly only after day 8, with mature oocytes first appearing around day 13, reaching full development (Stage 6). Subsequently, until day 20, vitellin accumulation remained slow or uneven.

$$Growth\;rate\;of\;length\;(\%)=\frac{L_{max}-L_{min}}{L_{min}}\times 100\%$$

$$Growth\;rate\;of\;weight\;(\%)=\frac{W_{max}-W_{min}}{W_{min}}\times 100\%$$

In this context, $L_{max}$ represents length of maximum ovariole, while $L_{min}$ denotes length of minimum ovariole measured within the same ovary. Correspondingly, $W_{max}$ and $W_{min}$ refer to the maximum and minimum body weight values observed among the measured specimens.

Regarding body weight, females from both dietary treatments reached their peak body weight at 10 days of age, and the pattern of weight gain from emergence to 20 days was generally consistent. Feeding on *C. cephalonica* eggs had no significant effect on female body weight (*t* = 1.347, df = 4, *p* = 0.249), indicating that *C. cephalonica* eggs can meet the nutritional requirements for basic somatic maintenance and growth in adult *S. japonicum*.

### 3.3. Effects of Feeding on C. cephalonica Eggs on the Expression of Reproduction-Related Genes in Female S. japonicum

**Figure 5:**
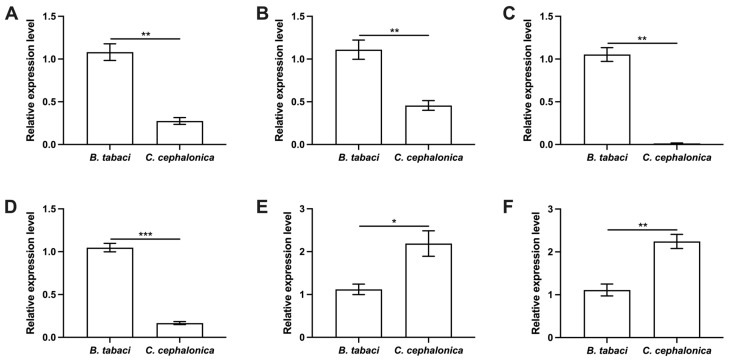
Effects of feeding on *C. cephalonica* eggs onthe expression of reproduction-related genes in *S. japonicum* females. (**A**) *Met*; (**B**) *JHAMT*; (**C**) *Vg*; (**D**) *VgR*; (**E**) *JHE*; (**F**) *Cu/Zn SOD*. All data are presented as mean ± standard error (SE) (*n* = 3). Asterisks indicate statistically significant differences: * *p* < 0.05, ** *p* < 0.01, *** *p* < 0.001.

To explore the molecular mechanisms underlying the impaired fecundity, we analyzed the expression levels of key reproduction-related genes (Figure 5). The qPCR results revealed distinct expression patterns in females fed on *C. cephalonica* eggs compared to those fed on *B. tabaci* nymphs. Specifically, the expressions of genes involved in juvenile hormone synthesis and signaling were significantly downregulated, including *JHAMT* (*t* = 5.176, df = 4, *p* = 0.007) and *Met* (*t* = 7.647, df = 4, *p* = 0.002). Similarly, genes related to vitellogenin synthesis and uptake were also suppressed: Vitellogenin (*Vg*) (*t* = 12.920, df = 2.021, *p* < 0.006) and Vitellogenin receptor (*VgR*) (*t* = 16.768, df = 4, *p* < 0.001). In contrast, the expression of genes involved in hormone degradation and antioxidant response was significantly upregulated: Juvenile hormone esterase (*JHE*) (*t* = 3.334, df = 4, *p* = 0.029) and Copper/zinc superoxide dismutase (*Cu/Zn SOD*) (*t* = 5.236, df = 4, *p* = 0.006).

## 4. Discussion

This study systematically evaluated the effects of *C. cephalonica* eggs as an alternative diet on the population fitness, ovarian development, and expression of reproduction-related genes in *S. japonicum* adults. The results indicated that while parental feeding on *C. cephalonica* eggs did not cause significant negative effects on the development, survival, or predatory capacity of their offspring, it significantly suppressed the reproductive capacity of the parental females. This was specifically manifested as a sharp prolongation of the pre-oviposition period and a substantial decrease in the total fecundity. The ovaries of coccinellids belong to the telotrophic type [27], and their maturation process involves two key stages: development of the ovarian framework and oogenesis, which are closely related to fecundity. Under conditions of abundant natural prey, the ovaries of most predatory ladybirds, such as *H. axyridis*, *Cryptolaemus montrouzieri* (Coleoptera: Coccinellidae), and *C. septempunctata*, can fully develop within 6–8 days [28,29,30] which is consistent with the results observed in *S. japonicum* fed on *B. tabaci* in this study (most matured by day 6). However, when feeding on non-natural prey, the fecundity of ladybirds is often affected. For instance, *H. axyridis* feeding on an artificial diet exhibited slower ovarian development compared to those fed aphids [15,31]. Furthermore, *D. catalinae* and *C. septempunctata* feeding on alternative prey also showed delayed ovarian development and retarded vitellogenin deposition, although they still reached sexual maturity [32,33]. Although feeding on *C. cephalonica* eggs did not affect the ovariole length or female body weight of *S. japonicum*, it significantly delayed the functional maturation of the ovaries. Specifically, the ovarian framework completed its development by day 6, although vitellogenin deposition did not commence until day 8, and the process was slow and discontinuous, with the earliest full maturation observed only by day 13. This delay and abnormality in vitellogenin accumulation are likely the direct causes of the prolonged pre-oviposition period and reduced fecundity.

Dietary conditions can influence trade-offs among life-history traits in insects, including resource allocation between reproduction and lifespan [34,35]. Different nutritional environments shape the strategic trade-offs of living organisms between investing in reproduction versus other physiological demands [36,37]. This is particularly evident for predators feeding on sub-optimal prey. Alternative prey can sustain predator survival during periods of natural prey scarcity, but predators on such diets often cease reproduction until food conditions improve sufficiently to support the high energetic costs of reproduction [38,39]. Under conditions of dietary shortage or unfavorable environments, physiological processes compete for limited energy resources, leading to trade-offs. The significantly extended lifespan and suppressed fecundity observed in female *S. japonicum* feeding on *C. cephalonica* eggs align with this “trade-off” strategy. Under conditions of limited resources or nutritional mismatch, organisms may reallocate their limited resources from reproduction to somatic maintenance and repair, thereby extending lifespan to survive unfavorable periods [34,40,41]. *C. cephalonica* eggs might not fully provide the nutritional signals required for optimal reproduction in *S. japonicum*, thus triggering this conservative energy allocation pattern, which trades reduced current fecundity for a longer lifespan. The physiological basis for such trade-offs can be multifaceted. Nutritional deficiency may be one factor. Indeed, diet quality is a key determinant of insect growth and reproduction [37,42]. *C. cephalonica* eggs might lack specific nutrients (certain sterols, lipids, or amino acids) essential for *S. japonicum* reproduction, or the ladybird may be unable to efficiently assimilate them. Another possibility is the absence of oviposition-stimulating chemical signals. Natural prey like *B. tabaci* may provide specific chemical cues necessary to trigger and sustain vitellogenesis and oviposition in *S. japonicum*, cues that are absent in *C. cephalonica* eggs. Similar mechanisms are known in other systems; for instance, the ectoparasitoid *Catolaccus grandis* (Hymenoptera: Pteromalidae) relies on physical contact with its host to stimulate egg maturation [43]. Additionally, a nutritionally imbalanced diet could induce systemic physiological stress, redirecting resources toward cellular maintenance and stress resistance pathways at the expense of reproductive investment.

Juvenile hormone (JH) has been demonstrated to play a regulatory role in reproduction in most insects, and its titer is maintained by a balance between synthesis and degradation processes [44]. This study revealed that feeding on *C. cephalonica* eggs led to significant downregulation of the expression of *Met* and *JHAMT* genes in female *S. japonicum*. *Met*, functioning as the JH receptor, initiates downstream signaling cascades upon binding with JH, regulating the synthesis and uptake of vitellogenin [45]. In *Aedes aegypti* (Diptera: Culicidae) and *P. japonica*, RNA interference of the *Met* gene resulted in arrested ovarian development and blocked vitellogenin deposition [46], which is highly consistent with the observed phenotypes of delayed ovarian development and slow vitellogenin accumulation in this study. *JHAMT* plays a crucial role in the final step of JH biosynthesis [47]. In *Bombyx mori* (Lepidoptera: Bombycidae), its gene expression positively correlates with JH biosynthesis [48], and RNAi-mediated knockdown of *JHAMT* expression in *Blattella germanica* (Blattodea: Blattellidae) resulted in a significant reduction in JH synthesis, accompanied by decreased vitellogenin expression in the fat body and a reduction in basal follicle length [49]. Conversely, the expression of *JHE* was significantly upregulated in female *S. japonicum* fed *C. cephalonica* eggs. *JHE* is the primary JH-degrading enzyme in most insects, capable of highly recognizing and hydrolyzing both JH-binding protein-bound and free JH into JH acid [50]. Enhancing or inhibiting its activity can disrupt JH levels in insects, affecting molting, pupation, and reproduction [51]. Therefore, the combination of reduced synthesis and enhanced degradation likely leads to a severe JH deficiency in *S. japonicum* fed *C. cephalonica* eggs. Simultaneously, the expression of both *Vg* and *VgR* genes was significantly reduced in these females. *Vg* is a crucial precursor for vitellogenin synthesis, and *VgR*-mediated endocytosis of Vg is essential for oocyte maturation. In *H. axyridis*, interference with *VgR* expression led to obstructed vitellogenin deposition and abnormal ovarian development [52]. In most insects, JH regulates the expression of the Vg gene in the fat body and influences the expression of the VgR gene in the ovaries, thereby affecting reproduction [53]. Consequently, the weakened JH signaling in *S. japonicum* fed *C. cephalonica* eggs leads to simultaneous impairments in both vitellogenin synthesis and transport, aligning with the anatomical observations of slow or uneven vitellogenin accumulation within the ovarioles. This is likely the reason for the prolonged pre-oviposition period and decreased fecundity. This molecular dysregulation could well be a downstream consequence of the nutritional or signaling deficiencies proposed above.

It is noteworthy that decreased JH levels are also closely associated with lifespan extension [54]. For example, in *Drosophila*, the insulin signaling pathway can regulate the aging process by influencing JH synthesis [55], and exogenous application of JH analogs, while enhancing early fecundity, shortens lifespan [56]. The results of this study suggest that the feeding on *C. cephalonica* eggs-induced attenuation of JH signaling, while suppressing reproduction, may also trigger physiological programs that extend lifespan. Furthermore, the expression of the *Cu/Zn SOD* gene was significantly upregulated in female *S. japonicum* fed *C. cephalonica* eggs. As a core enzyme in the defense against oxidative damage, the activity level of *Cu/Zn SOD* is closely related to lifespan regulation. In *Drosophila*, overexpression of *Cu/Zn SOD* extends lifespan [57,58], and long-lived termite queens also exhibit higher *Cu/Zn SOD* activity [59]. Therefore, the combined downregulation of JH signaling and upregulation of antioxidant defense may represent a coordinated physiological program. This program shifts the organism’s strategy from one of high reproductive investment to one favoring somatic persistence under suboptimal nutritional conditions. While our study provides correlative evidence at the gene expression level, further functional and omics-level studies are needed to fully validate this mechanistic model and elucidate the upstream regulatory networks.

## 5. Conclusions

We conclude that *C. cephalonica* eggs can support the juvenile development of *S. japonicum* but significantly suppress adult reproduction, making them a suboptimal sole diet for mass production. A pragmatic application strategy can be to use *C. cephalonica* eggs as a supplementary or maintenance diet, particularly for sustaining larval populations or during temporary shortages of the natural prey, *B. tabaci*. Future research on using mixed or sequential diets can mitigate the reproductive costs while leveraging the survival benefits of the alternative food.

## Figures and Tables

**Table 1:** Primer sequences.

Primer Name	Forward Primer Sequence	Reverse Primer Sequence
*JHAMT*	TTTGGATGTGGGATCAGGGG	TGGTGACACATCGACTGCAT
*Met*	TCGTACATAGGCGAGTTGGC	TCGAAGTGCGGCATGTTTTG
*JHE*	ACTGAACGCGACATCTGAGG	TGGGATCCTGTGGCGTAAAC
*Vg*	AGCCAATACCTCCGCAACAA	AGGATCACGAACAACGCAGT
*VgR*	AGTGGGCATTGCATTCCTGA	ACAAGCGCCTGATTTGCATC
*Cu/Zn SOD*	GGTGGACCAGCTGATGCTTT	CCTCTTGGAGCGCCAGATAA
*β-actin*	CGTACCACCGGTATCGTATTG	CGGAGGATAGCATGAGGTAAAG

**Table 2:** Length of ovariole and body weight of *S. japonicum* female adults (Mean ± SE).

Type of Prey	Length of Minimum Ovariole (mm)	Length of Maximum Ovariole (mm)	Growth Rate of Length (%)	Minimum Body Weight (mg)	Maximum Body Weight (mg)	Growth Rate of Weight (%)
*B. tabaci*	0.40 ± 0.01	1.56 ± 0.03	293.44 ± 5.10	10.40 ± 0.44	15.77 ± 0.18	52.24 ± 7.10
*C. cephalonica*	0.36 ± 0.01	1.26 ± 0.02	253.05 ± 14.77	11.37 ± 0.30	15.73 ± 0.38	38.78 ± 7.04
*p* value	0.003	<0.001	0.061	0.140	0.941	0.249

## Data Availability

The data presented in this study are available on request from the corresponding author.

## Abbreviations

The following abbreviations are used in this manuscript:

JH	Juvenile hormone
*Met*	methoprene-tolerant
*JHAMT*	Juvenile hormone acid O-methyltransferase
*JHE*	Juvenile hormone esterase
*Vg*	Vitellogenin
*VgR*	Vitellogenin receptor
*Cu/Zn-SOD*	Copper/zinc superoxide dismutase
